# Supplementary material for: A Global Screen for Assembly State Changes of the Mitotic Proteome by SEC-SWATH-MS
Source: Cell Syst. 2020 Feb 26;10(2):133–155.e6. doi: 10.1016/j.cels.2020.01.001 (PMC7042714; doi:10.1016/j.cels.2020.01.001)

Q8N5G0 | SMI20_HUMAN | SMIM20 C4orf52 MITRAC7
Monomer MW [kDa]: 7.702 Monomer expected elution fraction: 63

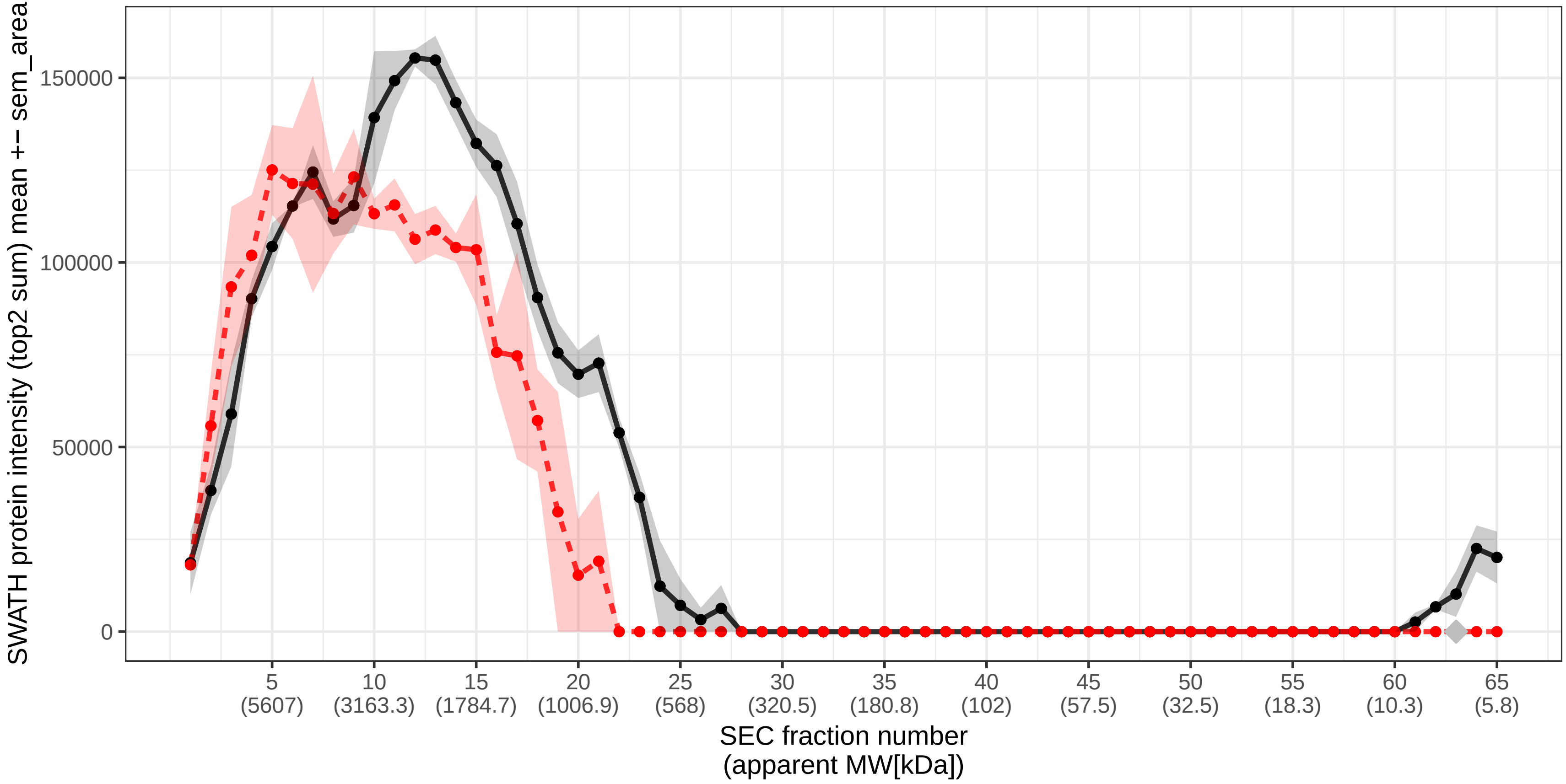

Supplement: Data S1. SEC-SWATH-MS Protein Chromatograms, Related to Figure 1 [file mmc6.zip › SECchrom_Q8N5G0_SMI20_HUMAN_SMIM20_C4orf52_MITRAC7.pdf]
